# Anemia in patients receiving anticancer treatments: focus on novel therapeutic approaches

**DOI:** 10.3389/fonc.2024.1380358

**Published:** 2024-04-02

**Authors:** Claudia Bozzini, Fabiana Busti, Giacomo Marchi, Alice Vianello, Claudio Cerchione, Giovanni Martinelli, Domenico Girelli

**Affiliations:** ^1^ Department of Medicine, Section of Internal Medicine, University of Verona, Verona, Italy; ^2^ EuroBloodNet Referral Center, Azienda Ospedaliera Universitaria Integrata Verona, Verona, Italy; ^3^ Istituto di Ricovero e Cura a Carattere Scientifico (IRCCS) Istituto Romagnolo per lo Studio dei Tumori (IRST) “Dino Amadori”, Meldola, Italy

**Keywords:** anemia, activin receptor ligand traps, cancer, chemotherapy, hepcidin, iron, therapy, prolyl hydroxylase inhibitors

## Abstract

Anemia is common in cancer patients and impacts on quality of life and prognosis. It is typically multifactorial, often involving different pathophysiological mechanisms, making treatment a difficult task. In patients undergoing active anticancer treatments like chemotherapy, decreased red blood cell (RBC) production due to myelosuppression generally predominates, but absolute or functional iron deficiency frequently coexists. Current treatments for chemotherapy-related anemia include blood transfusions, erythropoiesis-stimulating agents, and iron supplementation. Each option has limitations, and there is an urgent need for novel approaches. After decades of relative immobilism, several promising anti-anemic drugs are now entering the clinical scenario. Emerging novel classes of anti-anemic drugs recently introduced or in development for other types of anemia include activin receptor ligand traps, hypoxia-inducible factor-prolyl hydroxylase inhibitors, and hepcidin antagonists. Here, we discuss their possible role in the treatment of anemia observed in patients receiving anticancer therapies.

## Introduction

Anemia represents a common problem in cancer patients, affecting nearly 30-49% of those with solid tumors at diagnosis, further increasing to nearly 70% or more among those undergoing anticancer treatments or those with advanced disease (1–4). It is typically multifactorial, often involving different pathophysiological mechanisms in the same individual (5, 6). This makes treatment a difficult task (4), leaving a substantial fraction of patients sub-optimally or even untreated (1, 6). Although blood loss (e.g., in gastrointestinal tumors, or iatrogenic due to frequent blood sampling) and reduced red blood cell (RBC) survival can occur, decreased RBC production predominates in most patients. In turn, reduced erythropoiesis can be due to different mechanisms, which include: 1) direct bone marrow toxicity by anticancer drugs; 2) *absolute* deficiency of essential micronutrients (mainly, but not only, iron); 3) functional iron deficiency due to hepcidin-induced iron sequestration into macrophages; 4) impaired bone marrow response to erythropoietin (EPO) due to tumor-associated systemic inflammation; and bone marrow substitution by metastatic cancer cells (7, 8). This review focuses on anemia mainly related to anticancer treatments. For a comprehensive review of the pathophysiology of anemia in cancer patients, the readers are referred elsewhere (4).

The term Cancer-Chemotherapy Related Anemia (CCRA) underlines what it is often the major driver of anemia in cancer patients but cannot be viewed as an absolute entity. Rather, the coexistence of other mechanism(s) is often the rule, with important implications for the treatment of this condition (see below). Moreover, it has to be taken into account that CCRA is not a peculiarity of traditional chemotherapy agents, but can also occur as off-target effect with a number of newer antineoplastic agents (4, 9), including tyrosine kinase inhibitors (10), monoclonal antibodies (11), and immunomodulatory agents (12). Drug-induced kidney dysfunction can also contribute to hypoproliferative anemia through inadequate EPO production. Rarely immunotherapeutic agents known as checkpoint inhibitors (e.g., nivolumab, ipilimumab, and pembrolizumab) can induce antibody-mediated hemolytic anemia due to immune dysregulation (13). Overall, anemia remains one of the most frequent adverse effects of anticancer therapies, with a prevalence exceeding 90 percent for patients receiving certain treatments (3). The ECAS (European Cancer Anemia Survey) study, involving more than 15,000 patients, found an increasing prevalence of anemia from 32 percent in those newly diagnosed before receiving any treatment, to 44-51 percent in those receiving concomitant chemotherapy/radiotherapy or chemotherapy alone, respectively (1). Another survey in the U.S. estimated a prevalence of anemia of 61 percent in patients receiving chemotherapy, but only 25 percent of them received any anemia treatment (14). Very recently, the CARENFER study enrolling 1,221 patients with different types of solid malignancies, most of them (75.4 percent) treated with chemotherapy, found a high prevalence of iron deficiency (57.9 percent) with or without anemia (15), pointing out the need of better detection of this micronutrient deficiency in cancer patients. Prevalence and severity of anemia vary depending on the extent of the disease, the type, schedule, and intensity of treatment, and whether the patient has received prior radiotherapy and/or chemotherapy (16).

CCRA is associated with decreased functional capacity and a diminished quality of life (QOL) (16, 17), particularly because of its contribution to fatigue, which is commonly recognized as the most debilitating symptom (18). The degree of anemia in patients receiving active anticancer treatment is classified according to the National Cancer Institute (NCI) Common Terminology Criteria for Adverse Effects (CTCAE) (available at https://ctep.cancer.gov/protocoldevelopment/electronic_applications/docs/ctcae_v5_quick_reference_5x7.pdf), ranging from grade 1 or mild (Hb levels < 120/130 g/L in females/males but ≥100 g/L) to grade 4 (Hb levels < 80 g/L and symptoms suggesting a life-threatening condition) (for the detailed classification, see (4). Anemia can represent a dose-limiting toxicity that prevents patients from being treated with the full dose of chemotherapy. In patients with both solid and liquid malignancies, the development of severe anemia during the first cycle of chemotherapy is associated with an increased risk of dose delay and/or dose reduction in the subsequent chemotherapy cycle. Finally, evidence suggests that anemia may be an independent prognostic factor associated with reduced survival (19). For example, Waters and colleagues reported that 86 percent of 906 patients treated with chemotherapy for lung cancer at a single institution in the United States between 1999 and 2001 developed anemia during treatment (or within 1 month after completing) (20). The median survival rates of patients whose Hb levels were maintained above 12.0 g/dl were significantly (p < 0.001) higher than those of patients with lower Hb levels (20). However, because of the concurrence of multiple confounders, including transfusions, a direct causal relationship between anemia and survival in cancer patients has not yet been established (21).

## Therapeutic options for cancer-chemotherapy-related anemia

As for every type of anemia, elucidating the cause(s) is the cornerstone of an effective therapeutic approach. However, this is especially challenging in cancer patients. In its simplest form, CCRA can be the result of bone marrow toxicity in patients with early-stage cancer who were not anemic before starting chemotherapy. On the other hand, in patients with advanced disease, systemic inflammation, and micronutrient deficiencies (generally of iron, rarely of folate or B12 with the possible exception of low-middle income countries (22)) are often in the background, and chemotherapy merely unravels or exacerbates a pre-existing condition. In such cases the management should involve a multi-modal approach accounting for the severity of anemia, the associated inflammatory state, the impairment in iron metabolism, and the overall nutritional status.

Currently available treatments for anemia in patients receiving anticancer treatments include blood transfusions, iron supplementation, and erythropoiesis-stimulating agents (ESAs), like recombinant EPO (rhEPO) and darbepoetin-α. Each option has limitations, and guidelines restrict their use to specific settings (21, 23). Thereby, anemia in cancer patients is often poorly treated.

At present, we are witnessing an unprecedented development of novel anti-anemic drugs (24–26), many of them acting by ameliorating erythropoiesis. For this reason, since anemia in patients receiving anticancer treatments is essentially hypoproliferative due to bone marrow suppression, they could be theoretically of benefit also in this setting.

**Figure 1:**
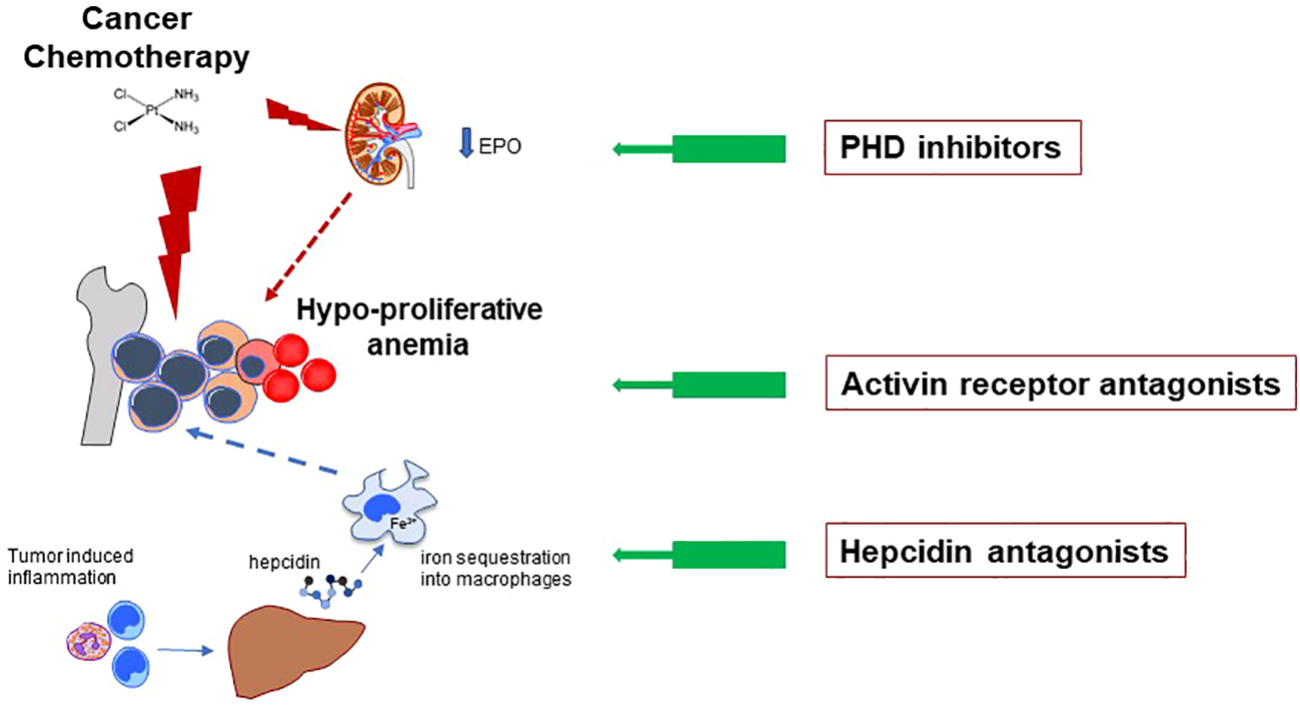
CCRA is essentially a hypoproliferative anemia mainly caused by toxic effects of anticancer drugs on rapidly proliferating RBC precursors in the bone marrow (major red lightning). However, the pathophysiology is often more complex, including drug-induced kidney damage with reduced production of endogenous EPO (minor red lightning), and low iron availability for erythropoiesis (dotted blue arrow). The latter can be due to either absolute or functional iron deficiency (FID, see main text). FID, in turn, is mainly determined by inflammatory-related increased hepcidin levels, leading to iron se-questration into macrophages. The general mechanisms of action of the innovative anti-anemic drug classes discussed in text are depicted. All the three classes inhibit path-way/molecule that modulate erythropoiesis, ultimately increasing RBCs production. Hypoxia Inducible Factor – Prolyl Hydroxylase inhibitors (HIF-PHIs) stabilize HIF leading to increased production of endogenous EPO. They represent an important alternative to the traditional administration of exogenous (recombinant) EPO. Moreover, they also have some favorable effects on iron metabolism (see main text). Activin Receptor (ACVR) ligand traps bind several members of the Transforming Growth Factor – beta (TGF-β) superfamily, thus reducing a key signaling pathway which negatively modulates late-stage erythropoiesis. Hepcidin antagonists increase iron availability for erythropoiesis.

Below we review the current and future options for anemia in patients receiving active anticancer treatments (**Figure 1**).

### RBC transfusions

Until the advent of ESAs in the 1990s, RBC transfusions—which were usually administered empirically when hemoglobin concentrations declined <100 g/L —were the only/primary treatment of cancer-related anemia. While transfusions are effective in providing an immediate increase in Hb, benefits are transient and risks are far from negligible, including anaphylactic reactions, transfusion-related acute lung injury (TRALI), circulatory overload, pathogens transmission, and increased susceptibility to infections possibly due to immunosuppressive modulation (27, 28). Of note, RBC transfusions have been independently associated with an increased risk for adverse outcomes in cancer patients undergoing surgery in terms of increased mortality, hospital length of stay, and tumor recurrence (29–31). Regarding CCRA, the Hb threshold for transfusion is not as clear as in other settings (32). The use of a liberal transfusion regimen (e.g., Hb threshold of 90 to 100 g/L) has not proven advantageous in terms of risk/benefit (33–35). A systematic review showed that restrictive transfusion regimens (e.g., Hb thresholds set at 70 to 80 g/L) in oncologic patients decrease blood utilization without increasing mortality and morbidity (36). The most recent guidelines issued by the Association for the Advancement of Blood and Biotherapies (AABB) in 2023 recommend a restrictive transfusion strategy in hemodynamically stable hospitalized adult patients with hematologic and oncologic disorders considering transfusion when the hemoglobin concentration is less than 70 g/L (37). However, the Authors recognize that the level of certainty evidence is low due to the few numbers of trials and patients enrolled. Thus, whether a rigorous restrictive regimen in cancer patients is as safe as a liberal regimen remains debated. *Ad hoc* studies investigating the optimal patient blood management strategy in CCRA are warranted. For the moment, especially in outpatients, an individualized approach putting first a comprehensive clinical judgment rather than mere laboratory abnormalities remains the best option (4).

### Iron supplementation

As mentioned above, iron deficiency (ID) in cancer patients is common and multifactorial (15, 38). For a comprehensive review of the numerous mechanisms possibly leading to ID in cancer patients, readers are referred elsewhere (6). Briefly, *absolute* ID (AID) corresponds to depletion of iron stores, which can be due, for example to bleeding in gastrointestinal cancer, malnutrition in patients with advanced disease, and malabsorption in patients submitted to wide bowel resections. Even more common in cancer patients is iron-restricted erythropoiesis, also sometimes referred to as *functional* ID (FID), which corresponds to insufficient iron availability despite theoretically adequate iron stores. Iron sequestration typically takes place in macrophages due to the upregulation of hepcidin (see below) by proinflammatory cytokines (8). Generally speaking, anemia in cancer patients can be reminiscent of the anemia of inflammation (39) when all other causes of anemia have been excluded. Finally, a particular type of FID can occur in CCRA patients treated with ESAs, where expanded erythropoiesis not infrequently outpaces iron delivery from stores, ultimately leading to ESAs unresponsiveness unless supplemental iron is given (40).

Indeed, numerous studies have proven the efficacy of intravenous (IV) iron in combination with ESAs in CCRA (41–43). An earlier systematic review and meta-analysis (performed in 2013) of randomized controlled trials comparing IV iron with no iron or oral iron in CCRA confirmed the efficacy of IV iron in combination with ESAs (44). The same meta-analysis also reported the efficacy of IV iron alone, while only a few smaller trials were available at that time (44). Indeed, with the recent advent of modern third-generation IV iron preparations (45), evidence is accumulating on the efficacy of iron monotherapy in CCRA (46–49). CCRA patients with concomitant AID are the most suitable candidates for this approach, but diagnosing AID in cancer patients using traditional iron biomarkers is challenging (6), due, for example, to ferritin upregulation by tumor necrosis factor-alpha (TNF-a) and pro-inflammatory cytokines (50, 51). To identify patients that could benefit from iron supplementation, different ferritin cut-offs have been proposed, ranging from 100 (21) to up to 800 (52) μg/L, also depending on the presence or absence of concomitant low transferrin saturation (TSAT <20%). However, a consensus is still lacking, and there is an urgent need for better biomarkers (8). The most promising one in this sense is hepcidin, which has the potential to distinguish absolute iron deficiency even in inflammatory conditions (53). Indeed, at least three independent studies have shown its usefulness in predicting AID (and hence the response to IV iron) in CCRA (48, 54, 55). Nevertheless, due to the lack of harmonization between different valuable assays (56, 57), a universal cutoff is not yet available. On the other hand, there is substantial agreement on the little usefulness of oral iron in the majority of CCRA patients due to several caveats, including side effects (e.g., gastrointestinal discomfort), *poor compliance*, especially in patients on polypharmacy, and inflammation-induced malabsorption through increased hepcidin (4–6, 9). Iron administration in CCRA should be prescribed to patients within the framework of currently available guidelines (21, 52).

### Erythropoiesis stimulating agents

Since their appearance in the 1990s, erythropoiesis-stimulating agents (ESAs) substantially changed the scenario of CCRA treatment. The efficacy of either rhEPO (epoetin) or the analog darbepoetin in reducing transfusions and improving QoL has been documented by several studies and meta-analyses (58–60). Biosimilars are available, showing acceptable interchangeability (61). However, ESAs in cancer patients have raised major concerns, including increased risk of thrombosis (62) and decreased survival possibly due to tumor progression (63). The latter could be due to off-target ESAs binding to ephrin-B4 receptors, which in turn increase angiogenesis and tumor neovascularization (64). This has led to consistent restriction of ESAs use for treating anemia in cancer patients. According to recent guidelines jointly issued by the American Society of Hematology and the American Society of Clinical Oncology (for a comprehensive review, see (23), these drugs should be reserved for CCRA patients receiving chemotherapy with palliative intent and who are expected to have short survival. Outside the scenario of CCRA, ESAs are currently used in patients with low-risk myelodysplastic (MDS) syndromes.

### Novel anti-anemic drugs with a potential use for CCRA

#### Activin type II receptor (ACVR) ligand traps

This novel class includes two drugs, luspatercept and sotatercept. They are recombinant fusion proteins that act by inhibiting negative regulators of late-stage erythropoiesis, like activin B and other transforming growth factor beta (TGF β) superfamily ligands (65–70). Thus, they can be viewed as molecular traps that facilitate erythropoiesis by removing biological brakes to the process (24, 26, 71). At variance with EPO, which stimulates the proliferation of RBC progenitors during the early stages of erythropoiesis, ACVR ligand traps distinctly favor late-stage erythropoiesis. Their effect, also through modulation of marrow stromal cells (66), is prevalent in differentiation rather than proliferation of RBC progenitors. This explains why these compounds work particularly well in conditions of pathologically expanded but ineffective erythropoiesis, like thalassemic syndromes and low-risk MDS (see below).

Of note, sotatercept was initially developed to increase bone mineral density in malignant bone disease and in osteoporosis (72, 73), with the unexpected observation of an erythroid response (73, 74). Later, the same effect was observed with the analog luspatercept (75). Improvement of ineffective erythropoiesis was subsequently confirmed in murine models of b-thalassemia (69). This recently translated into one of the major achievements in the treatment of patients with beta-thalassemic syndromes, where luspatercept has been proven to increase Hb and substantially decrease transfusion needs (76). Indeed, luspatercept has been preferentially developed in clinical studies because of a higher specificity activin B (rather than for activin A) as compared to sotatercept, with a lower probability of determining off-target effects (77). The sustained effect on erythropoiesis has also prompted the use of luspatercept for anemia associated with hematologic malignancies (77, 78). Low-risk MDS have been selected because of the need for alternative strategies to frequent RBC transfusions in anemic patients with prolonged anticipated survival. The MEDALIST trial enrolled 229 transfusion-dependent patients affected by MDS with ringed sideroblasts, who were either refractory or not candidates for ESAs because of high baseline EPO level (79). Transfusion independence for ≥8 weeks was observed in 38 percent of the patients in the luspatercept group, as compared with 13 percent in the placebo group (*P*<0.001). Such positive results have been recently confirmed by the COMMANDS trial, which enrolled 356 patients randomized to receive either luspatercept or epoetin alfa and showed a higher rate of transfusion independence in the luspatercept group (80). Luspatercept shows a favorable safety profile (79), and is now approved for low-risk MDS by both FDA and EMA. A post-hoc analysis of the MEDALIST study provided support for the efficacy of Luspatercept also in a subgroup of patients affected by myelodysplastic syndromes/myeloproliferative neoplasm with ring sideroblasts and thrombocytosis (MDS/MPN-RT-T) (81); Luspatercept is currently under active investigation for other types of anemia in hematological malignancies, like myeloproliferative neoplasm-associated myelofibrosis (NCT03194542). Preliminary results of the latter study have been recently presented showing that transfusion-independency was reached in nearly one-third of patients on ruxolitinib that were initially transfusion dependent (82). Such promises and successes in anemia associated with hematological malignancies make ACVR ligand traps an attractive option also for anemia in other malignancies. To date, only limited/incomplete data are available for sotatercept in CCRA. Two Phase II placebo-controlled studies evaluated this anti-anemic drug in patients with metastatic breast cancer receiving myelosuppressive chemotherapy or with advanced/metastatic solid tumors receiving platinum-based chemotherapy (83). Unfortunately, both studies were terminated early due to slow patient accrual. Nevertheless, preliminary results indicated that mean hemoglobin levels increased ≥10 g/L in up to 66.7 percent of patients receiving sotatercept, as compared to 0 percent in placebo groups (83). In both studies, the safety profile was comparable to that of placebo groups. While caution is needed in interpretation due to the small populations studied, both studies point out the potential benefit of ACVR ligand traps for CCRA.

#### Hepcidin antagonists

As mentioned above, hepcidin overexpression plays a major role in the anemia of inflammation (39, 84) and often contributes to anemia in cancer patients by reducing iron availability for erythropoiesis because of iron sequestration in cells, mainly in macrophages (39). This condition of FID can be, in principle, reversed by counteracting hepcidin activity (85). However, inhibiting hepcidin is not as simple as it could be theoretically anticipated. This hormone, which represents the master regulator of iron homeostasis (53), is continuously produced by the liver in discrete amounts (nM concentration). Direct hepcidin binders have been developed (85, 86), including a fully humanized monoclonal antibody (LY2787106), and a structured mirror-image RNA oligonucleotide (NOX-H94) (87), both with high affinity for human hepcidin. A phase 1 multicenter trial evaluated LY2787106 in 33 patients with different malignancies and anemia, 19 of whom received chemotherapy (88). LY2787106 was well-tolerated and induced initially dose-dependent increases in serum iron and transferrin saturation in patients with high hepcidin concentration. However, iron parameters quickly returned to baseline (within 1 week), and the primary efficacy endpoint (increased Hb level) was not reached (88). No further development of the drug has appeared in the literature.

NOX-H94 was initially evaluated in human volunteers subjected to experimental endotoxemia by lipopolysaccharide administration, with an observed increase of serum iron consistent with hepcidin inhibition (89). The drug was further shown to effectively inhibit hepcidin in a dose-dependent manner and was apparently well-tolerated (90). In 2014, a phase-II pilot study on 12 patients with anemia and hematological malignancies reported increased hemoglobin levels in 5 (91). However, again, no further developments have been reported. Another crucial point that makes hepcidin antagonization far from simple is represented by the substantial complexity of molecular regulation of hepcidin production (92, 93). Many strategies alternative to direct hepcidin neutralization have been reported, including antagonists of the bone morphogenetic protein 6 (BMP6) pathway with heparin derivatives (94–97), anti-BMP6 antibodies (98), dorsomorphin (99), and ferroportin stabilizers (100, 101). Further drugs that modulate the hepcidin-ferroportin axis include anti-hemojuvelin (HJV) antibodies (currently under evaluation in two clinical trials NCT05745883 and NCT05320198), and momelotinib. This latter drug is a promising and potent inhibitor of the ACVR1/ALK2 receptor, which has been developed for the treatment of anemia in patients with myelofibrosis (102–104). In a broad sense, numerous other drug classes indirectly regulate hepcidin (for a recent review, see (105)). Among them are interleukin 6 (IL-6) and interleukin-1 beta (IL-1b) inhibitors, signal transducer and activator of transcription 3 (STAT3) inhibitors, and Hypoxia-inducible factor–prolyl hydroxylase inhibitors (HIF-PHIs) (see below). While several clinical trials are underway (90), no data are available for patients with CCRA.

#### Hypoxia-inducible factor-prolyl hydroxylase inhibitors

Hypoxia-inducible factors (HIFs) are transcription factors that mediate the cellular response to hypoxic conditions via the upregulation of several genes that are key to erythropoiesis, including those for EPO, the EPO receptor, and even for some critical proteins involved in iron metabolism (25, 106, 107). HIFs are heterodimers consisting of a constitutively expressed HIF-1β subunit and an O2-regulated HIF-α subunit. Under normoxic conditions, HIF is degraded by the hydroxylation of prolyl residues of the HIF-α subunit by HIF prolyl hydroxylases (PH). By preventing HIF degradation, HIF-PHIs stimulate endogenous EPO and promote erythropoiesis (25). Several HIF prolyl hydroxylase inhibitors have been developed and successfully used to treat anemia in chronic kidney disease (CKD) patients (108).

HIF-PHIs induce endogenous EPO at a concentration near the physiological range, representing a substantial difference with peaks determined by rhEPO administration and may explain the lower rate of cardiovascular adverse events. Other advantages over traditional ESAs include oral administration, lower costs, and lower immunogenicity. As mentioned above, it is worth noting that HIF-PHIs have pleiotropic favorable action on erythropoiesis beyond EPO stimulation (25). Indeed, critical players in iron metabolism like the Transferrin Receptor, Divalent Metal Transporter 1, and Ferroportin (the cell membrane receptor of hepcidin) are controlled by HIF through binding to their hypoxia-responsive elements, resulting in potent transcriptional activation (109–111). In this way, HIF-PHIs improve iron absorption from the gut and iron uptake and utilization by erythroid precursors through upregulation of transferrin receptor, ultimately leading to decreased levels of hepcidin, ferritin, and transferrin saturation (112). Roxadustat, one of the main HIF-PHIs already approved for anemia in CKD (113, 114), is currently being investigated in oncologic patients with anemia. MATTERHORN (NCT03263091) is a phase 3, randomized, double-blind, placebo-controlled study to assess the efficacy and safety of roxadustat in anemia of lower risk-MDS. In the dose-selection stage, Roxadustat was well-tolerated, and 37.5% of pts (9/24) with LR-MDS and low RBC transfusion burden achieved transfusion independence (TI) (115). In the MATTERHORN double-blind stage, a more significant percentage of pts in the Roxadustat arm compared with the placebo arm were TI responders (47.5% vs. 33.3%). However, this difference did not reach statistical significance (*p*=0.22) (116). A recent phase 2 open-label trial has investigated the efficacy and safety of Roxadustat for CCRA in various non-myeloid malignancies, including pancreas, breast, lung, and ovarian cancer, who have planned concurrent myelosuppressive therapies (117). Subgroup analyses demonstrated the efficacy of Roxadustat in correcting Hb regardless of tumor type and chemotherapy received. Adverse events were consistent with observations in patients with advanced-stage malignancies.

Despite their undoubtful benefit in the short-term correction of anemia, concerns have been raised about the possible off-target effects of HIF-PHIs during long-term use. Indeed, HIFs are ubiquitously expressed and regulate a broad spectrum of cell functions beyond erythropoiesis. Theoretically, HIF-PHIs may foster latent cancers since HIF activation in a hypoxic environment could promote cell proliferation and angiogenesis. This calls for caution, especially in older people. Nevertheless, no data thus far have revealed any effect of HIF-PHIs on tumor progression. On the other hand, chronic hypoxia caused by prolyl hydroxylase inhibition may inactivate cancer-associated fibroblasts, leading to decreased tumor metastatic spread. Ideally, future compounds able to selectively stimulate HIF2 could reduce undesirable off-target effects.

## Conclusions and perspectives

Anemia in cancer represents a significant unmet clinical need because of its high prevalence, the impact on QoL, and the likely implications on survival. Currently available treatments, particularly RBC transfusions and ESAs, have significant limitations. This results in a substantial proportion of patients left untreated or treated inadequately (4, 21).

The first step of the treatment of anemia in these patients is disentangling the causes, particularly those that can be readily treated, like iron deficiency, which is highly prevalent in cancer patients (3, 14) and relatively simple to correct with the newer intravenous iron preparations.

Nowadays, the therapeutic armamentarium of anti-anemic drugs is rapidly expanding, with remarkable successes in conditions like CKD, thalassemic syndromes, and certain MDS. For the moment, only a minority of the agents belonging to the most promising classes shown in **Table 1** are being specifically investigated in CCRA. Each of them shows promise and caveats.

**Table 1:** Novel anti-anemic drugs that could be used for Cancer Chemotherapy-Related Anemia.

Class	General anti-anemic mechanism	Molecular mechanism	Examples	Comments
**Hepcidin antagonists**	Increased iron availability to BM erythroid precursors	Inhibition of hepcidin by direct binding or by interfering with its action	LY2787106 NOX-H94 Heparin derivatives Anti-BMP6 agents Ferroportin stabilizers	Promising, but only in pre-clinical models for the moment
**Hypoxia Inducible Factors-Prolyl Hydroxylase inhibitors (HIF) stabilizers**	↑ endogenous EPO ↑ iron utilization	Inhibition of HIF prolyl-hydroxylase. ↑ expression of key iron-related genes	Roxadustat Vadadustat Daprodustat Molidustat	Oral administration; possible better CV risk profile compared to ESAs; concerns about off-target effects during long term use
**Activin receptor IIA ligand traps**	Stimulation of erythropoiesis	Molecular trapping of TGF-β superfamily inhibitors of late-stage erythropoiesis	Luspatercept Sotatercept	Already approved for anemia in certain hematologic malignancies (e.g., low-risk MDS) s.c. administration every 3-4 weeks

BM, bone marrow; EPO, erythropoietin; CV, cardiovascular; ESAs, erythropoiesis stimulating agents; MDS, myelodysplastic syndrome; CCRA, Cancer-Chemotherapy Related Anemia.↑, increased.

Nevertheless, the multifactorial pathogenesis of anemia in cancer, which can also change during the evolution of the disease, makes it unlikely that a single drug will be successful in the heterogeneous population of anemic cancer patients. Rather, an individualized holistic approach, including a thorough evaluation of nutritional status and, possibly, the combined use of more than one drug/supplement targeting different steps of erythropoiesis, should be implemented to provide an effective treatment that improves survival and quality of life of anemic cancer patients.

## Author contributions

CB: Writing – original draft, Writing – review & editing. FB: Writing – review & editing. GiaM: Writing – review & editing. AV: Writing – review & editing. CC: Writing – review & editing. GioM: Writing – review & editing. DG: Writing – original draft, Writing – review & editing.
